# Scyl1 Regulates Golgi Morphology

**DOI:** 10.1371/journal.pone.0009537

**Published:** 2010-03-04

**Authors:** Jonathon L. Burman, Jason N. R. Hamlin, Peter S. McPherson

**Affiliations:** Department of Neurology and Neurosurgery, Montreal Neurological Institute, McGill University, Montreal, Quebec, Canada; The University of Queensland, Australia

## Abstract

**Background:**

Membrane trafficking is a defining feature of eukaryotic cells, and is essential for the maintenance of organelle homeostasis and identity. We previously identified Scy1-like 1 (Scyl1), a member of the Scy1-like family of catalytically inactive protein kinases, as a high-affinity binding partner of COPI coats. COPI-coated vesicles control Golgi to endoplasmic reticulum trafficking and we observed that disruption of Scyl1 function leads to a decrease in trafficking of the KDEL receptor via the COPI pathway. We reasoned that if Scyl1 plays a major role in COPI trafficking its disruption could influence Golgi homeostasis.

**Methodology/Principal Findings:**

We performed Scyl1 knock down in cultured cells using previously established methods and observed an alteration in Golgi morphology. Both the surface area and volume of the Golgi is increased in Scyl1-depleted cells, but the continuity and polarity of the organelle is unperturbed. At the ultrastructural level we observe a decrease in the orderly structure of the Golgi with an increase in cisternal luminal width, while the number of Golgi cisternae remains unchanged. The golgin family of proteins forms a detergent resistant network that controls Golgi homeostasis. Disruption of this protein network by knock down of the golgin p115 disrupts the Golgi localization of Scyl1. Moreover, we find that Scyl1 interacts with 58K/formiminotransferase cyclodeaminase (FTCD), a protein that is tightly associated with the cis face of the Golgi.

**Conclusions/Significance:**

Our results place Scyl1 at an interface between the golgin network and COPI trafficking and demonstrate that Scyl1 is required for the maintenance of Golgi morphology. Coupled with the observation from others that Scyl1 is the gene product responsible for the neurodegenerative mouse model mdf, our results additionally implicate the regulation of COPI trafficking and Golgi homeostasis in neurodegeneration.

## Introduction

It is estimated that the entire surface area of the plasma membrane of a fibroblast passes through its secretory pathway every 3 hours [1], making it imperative that these cells stringently regulate membrane flow in order to maintain organelle identity. A major function for coated vesicle systems in eukaryotes is the maintenance of organelle homeostasis in the face of such massive flux [1]. The molecular basis of organelle homeostasis has been extensively investigated, with many studies focusing on the Golgi apparatus since the Golgi is a central trafficking hub [2]. One factor that contributes to the maintenance of Golgi structure is the balance of input and output from membrane trafficking pathways, and increases in anterograde membrane flux by overexpression of the vesicular stomatitis G-protein (VSVG) secretory cargo has been shown to increase the size of the Golgi apparatus [3]. Logically, a decrease in retrograde trafficking would be predicted to result in an expanded Golgi.

Two important cellular systems that regulate Golgi homeostasis are COPI-coated vesicles and the golgin family of molecules [4], [5]. The COPI vesicle system is an essential component for trafficking in the early secretory pathway, functioning within the Golgi stack and in a Golgi to endoplasmic reticulum (ER) retrograde pathway [5], [6]. COPI coats bud vesicles containing Golgi resident proteins, proteins that cycle between the ER and the Golgi, and ER resident proteins that have escaped from the ER [6], [7]. COPI trafficking is essential for cell growth and survival, as well as the maintenance of Golgi structure [8], [9] and knock down of the βCOP subunit of the COPI coat in HeLa cells results in an increase in Golgi volume and a fragmented Golgi [10]. Still, the total complement of molecules involved in COPI trafficking and their relationship to each other and to Golgi homeostasis is unclear [11].

Golgins are a class of Golgi-associated proteins that have been ascribed numerous functions in the cell including acting as tethers for incoming vesicles, laterally linking Golgi cisternae and regulating Golgi inheritance during mitosis [5]. Golgins are peripheral membrane proteins containing extensive coiled-coil domains. They have a detergent resistant interaction with Golgi membranes and undergo exchange between their Golgi-associated and cytosolic pools. Golgins also interact with each other and with components of coated vesicle trafficking systems. In particular, golgins interact extensively with Rab GTPases, and through these interactions form a protein network that facilitates vesicle-docking events [5], [12]. For example, the golgins GM130 and p115 have been identified as part of the golgin network, and both regulate COPI trafficking [13]. It has also been shown that p115 and GM130 are components of a subset of COPI vesicles that contain KDEL receptor, which is involved in the retrieval of ER resident proteins and p115 has been shown to bind to βCOP, while GM130 is known to interact with the small GTPase Rab1 [10], [13], [14]. Therefore, COPI-associated golgins are linked to retrograde trafficking from the Golgi to the ER.

Scy1-like 1 (Scyl1) is a member of the Scy1-like family of catalytically inactive protein kinases [15]. In a previous study we demonstrated that Scyl1 binds COPI coats with high affinity and localizes to the ER/Golgi intermediate compartment (ERGIC) where it co-localizes with ERGIC53 and COPI [16]. Scyl1 also localizes to the cis-Golgi where it co-localizes with GM130 and 58K/formiminotransferase cyclodeaminase (FTCD) [16]. 58K is a peripheral membrane protein that demonstrates golgin characteristics, being tightly associated with the cis-Golgi and displaying a similar extraction profile as GM130 [17]. Knock down of Scyl1 disrupts KDEL receptor trafficking from the Golgi to the ER without affecting the anterograde trafficking of GFP-VSVG [16]. In the current study we demonstrate that Scyl1 regulates Golgi homeostasis. Knock down of Scyl1 causes the Golgi to enlarge and there is a decrease in the orderly structure of the organelle. In addition, we reveal that Scyl1 interacts with 58K and requires an intact golgin network for its association with the Golgi. Although Scyl1 shares several properties with other golgins, knock down of Scyl1 leads to an expanded but intact Golgi, whereas knock down of either COPI or golgins cause the Golgi to expand and fragment. These data place Scyl1 at a unique interface between the golgin network and COPI trafficking, and demonstrate a role for Scyl1 in the regulation of Golgi homeostasis. As Scyl1 loss of function is causative of the neurodegenerative disease model mdf, our results link alterations in Golgi homeostasis to neurodegeneration.

## Results

### Scyl1 Regulates Golgi Homeostasis

**Figure 1:**
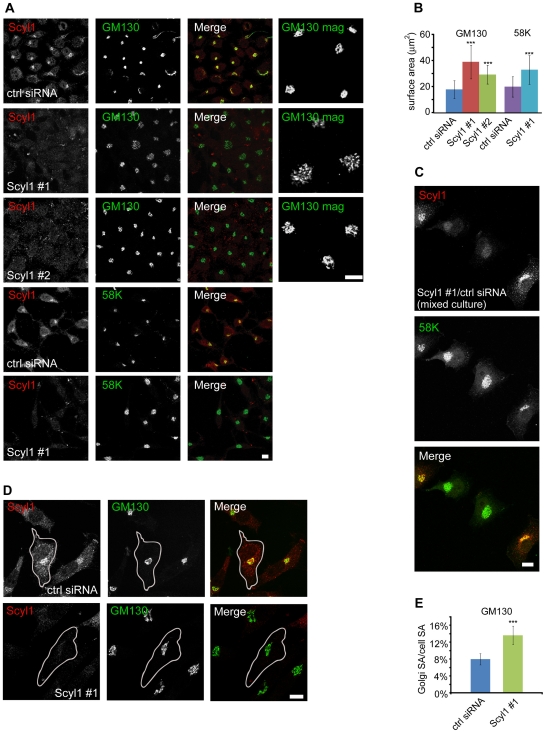
Expansion of the Golgi following Scyl1 knock down. **A,** HeLa cells were transfected with control (ctrl) siRNA or one of two previously characterized siRNAs (Scyl1 #1 and #2) specific for Scyl1 [16]. The cells were subsequently fixed and processed for indirect immunofluorescence with antibodies against Scyl1 (red) and either GM130 or 58K (green). Magnified views of regions of the GM130 staining are shown in the rightmost 3 panels. The scale bars = 10 µm. **B,** The two-dimensional surface area was calculated for a large number of Golgi as described under Materials and Methods. Despite variability in Golgi size as indicated by the overlapping error bars, which indicate standard error of the mean, Scyl1 knock down causes a significant increase in the surface area of the Golgi (*** p<0.001) as determined by a Student's t-test. **C,** Cells treated with ctrl siRNA or Scyl1 #1 siRNA were subsequently mixed and co-stained for Scyl1 (red) and 58K (green). The two cells in the middle, lacking Scyl1 expression have expanded Golgis. The scale bar = 10 µm. **D,** HeLa cells, transfected with control siRNA or Scyl1 #1 siRNA were subsequently fixed and processed for indirect immunofluorescence with antibodies against Scyl1 (red) and GM130 (green). Outlines of the cells are indicated. The scale bar = 10 µm. **E,** The two-dimensional surface area of the Golgi was calculated and presented as a percentage of the two-dimensional whole cell area. The Golgi in Scyl1 knock down cells covers a significantly (*** p<0.001, Student's t-test) larger area of the cell. Error bars represent standard error of the mean for all graphs.

While examining the role of Scyl1 in membrane trafficking we observed alterations in Golgi morphology. HeLa cells, which have robust Scyl1 expression were transfected with either control siRNA or one of two previously characterized Scyl1-specific siRNAs, Scyl1 #1 and #2 [16]. Scyl1 #1 and #2 strongly reduce Scyl1 levels without influencing the levels of a number of control proteins including βCOP and GM130 [16]. Four days post-transfection cells were fixed and co-stained for Scyl1 and the cis-Golgi markers GM130 and 58K (Figure 1A). Low power examination of large fields of cells reveal that under control conditions, both 58K and GM130 have a juxtanuclear Golgi localization that appears enlarged or expanded following Scyl1 knock down (Figure 1A). This is better appreciated in higher magnification views of the GM130 staining (Figure 1A). To quantify this result the two-dimensional surface area of individual Golgis stained for 58K or GM130 were calculated for large numbers of cells over 3 separate experiments (see Materials and Methods for a description of the quantification). A significant increase in the surface area of the Golgi revealed with GM130 staining was observed in cells depleted of Scyl1 using both siRNAs although Scyl1 #1 treated cells showed a larger increase (Figure 1, A and B), consistent with the fact that Scyl1 #1 causes a more dramatic knock down [16]. A significant increase in Golgi surface area was also detected by 58K staining in Scyl1 #1 (Figure 1, A and B) and Scyl1 #2 (data not shown) treated cells. These data strongly suggest that the increase in the two-dimensional surface area of the Golgi is specific for Scyl1 knock down. Knock down status for all cells included in the quantification were confirmed by co-staining with Scyl1 and there was no change in the overall fluorescence intensity of GM130 or 58K between control and knock down cells (data not shown). Thus, Scyl1 appears to play a role in the regulation of Golgi morphology.

To ensure that we were not missing significant Golgi structure outside of the ∼0.2 µm focal plane, we acquired Z-series confocal images of mixed cultures of HeLa cells treated with either control or Scyl1 siRNA #1. This protocol allowed for imaging of control and Scyl1-depleted cells in the same field, thus assuring that the confocal parameters, cell culture and staining conditions were identical within the sample image. Co-staining with 58K and Scyl1 demonstrated that cells depleted of Scyl1 exhibited an expanded Golgi throughout the volume of the cell (Figure 1C and Movie S1).

Because larger cells have, on average, a larger Golgi, we tested for contributions of cell size on the expansion phenotype by quantifying the two-dimensional surface area of the Golgi as well as the area of the full cell in control and Scyl1-depleted conditions (Figure 1, D and E). The Golgi surface area was significantly increased relative to the surface area of the cell in Scyl1-depleted cells. While the Golgi occupied 7.95% of the cell in control, it occupied 13.63% of the cell following Scyl1 knock down (Figure 1, D and E). We also immunostained Scyl1-depleted and control HeLa cells with the ER marker calnexin, the microtubule marker β-tubulin and a marker of clathrin-coated vesicles, AP-2. Scyl1 knock down did not cause an obvious change to any of these subcellular structures (data not shown).

### The Golgi Remains Intact but with an Increased Volume Following Scyl1 Knock Down

**Figure 2:**
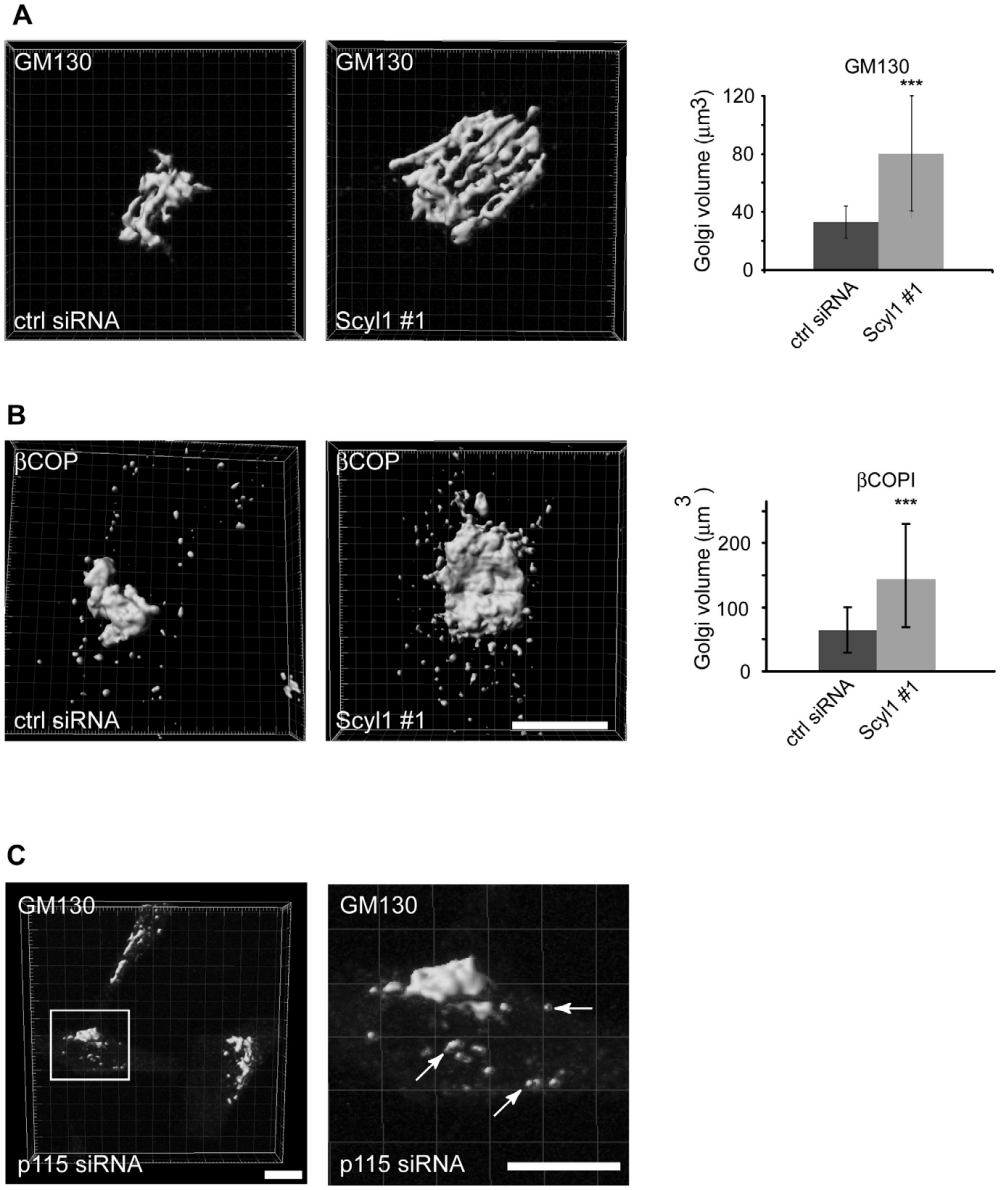
The volume of the Golgi is increased following Scyl1 knock down. **A,** HeLa cells transfected with control (ctrl) or Scyl1 #1 siRNA were stained for GM130. Z-series of complete Golgi stacks were imaged by confocal microscopy and volume surface rendered using Imaris software as described in Materials and Methods. The scale bar = 10 µm. The volume of the Golgi labelled with antibody against GM130 was determined as described in Materials and Methods. A significant increase (*** p<0.001; Student's t-test) in Golgi volume was seen, as indicated graphically on the right. **B,** HeLa cells transfected with control or Scyl1 siRNA were stained for βCOP and processed as described in **A**. **C,** HeLa cells transfected with p115 siRNA were stained for GM130. Z-series of complete Golgi stacks were imaged by confocal microscopy and volume surface rendered using Imaris software. The scale bar = 10 µm. Error bars represent standard deviation.

To determine if Scyl1 regulates the volume of the Golgi, control and Scyl1 knock down cells were stained with βCOP or GM130. The expression levels of these proteins are not altered by Scyl1 knock down [16]. Z-series of complete Golgi stacks were imaged by confocal microscopy. Imaris software was used to volume surface render the confocal stacks and to quantify Golgi volume (see Materials and Methods for a description), revealing a significant increase in the volume of the Golgi in Scyl1-depleted cells (Figure 2, A and B). Under control conditions, the Golgi averaged 34.8+/−11.3 µm^3^ as determined with GM130 staining (Figure 2A) and 66.3+/−37.1 µm^3^ as determined for βCOP staining (Figure 2B), while Scyl1 knock down resulted in Golgi volumes of 80.3+/−40.4 µm^3^ for GM130 (Figure 2A) and 148.5+/−82 µm^3^ for βCOP (Figure 2B). The difference between the average Golgi volume for GM130 and βCOP likely reflects the fact that COPI localizes to the entire Golgi while GM130 is localized only to the cis-Golgi. Our observed measurements for Golgi volume obtained by confocal three-dimensional reconstruction and volume rendering are in accordance with previous estimates using EM-based techniques. For example Ladnisky et al. [18] used EM tomography and assessed a Golgi volume of ∼60–80 µm^3^ from a rat kidney NRK cell, while earlier EM sterological studies of developing rat livers demonstrated a Golgi volume of between 80 and 240 µm^3^, depending on the developmental stage [19].

The approximately 2-fold increase in Golgi volume in Scyl1-depleted cells reported here is similar to that found previously in βCOP knock down conditions [10]. However, whereas depletion of βCOP expands and fragments the Golgi [10], three-dimensional reconstruction of control and Scyl1-depleted cells revealed that expanded Golgis were seemingly intact (Figure 2, A and B and Movies S2 and S3). This was further emphasized by comparison to cells in which the golgin p115 was knocked down using a previously established siRNA [20]. p115 knock down resulted in a fragmented Golgi structure (Figure 2C), in accordance with previous results [20]. Thus, Scyl1 knock down leads to a Golgi expansion phenotype but the Golgi itself appears intact. Since knock down of both βCOP and the golgin p115 lead to a fragmented Golgi, our results highlight a unique role for Scyl1 in regulation of Golgi morphology.

### Golgi Integrity and Polarization Is Normal in Scyl1-Depleted Cells

**Figure 3:**
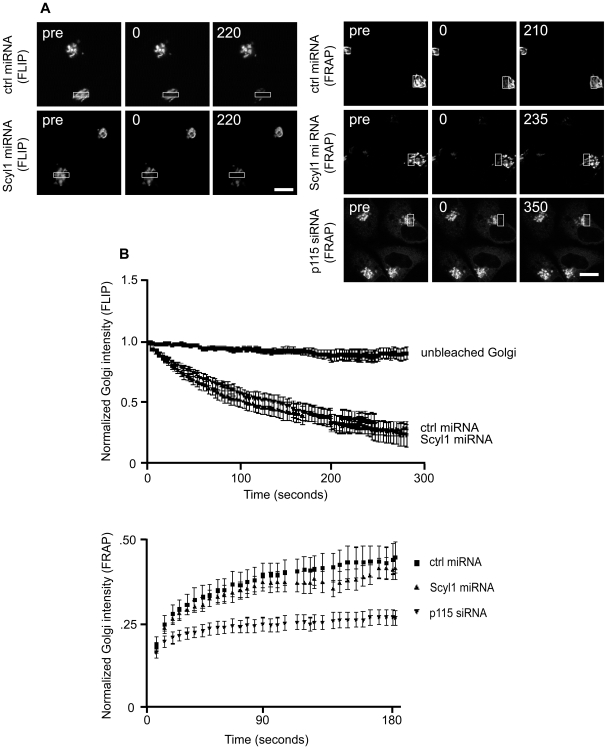
The expanded Golgi remains intact. **A,** HeLa cells transfected with control (ctrl) or Scyl1 miRNA and MannII-GFP were subjected to FRAP and FLIP analysis as described in the Materials and Methods. The images reveal MannII-GFP fluorescence with boxes indicating the photobleached areas. Images were collected prior to bleaching (pre), immediately after the first bleach (0) or at the indicated times (in seconds) after bleaching. Scale bars = 10 µm. **B,** The normalized intensity of fluorescence staining over the course of the FLIP and FRAP experiments. For the FLIP experiments (top graph) the y-axis value reflects the % GFP intensity normalized to the zero time fluorescence intensity prior to the repeated photobleachings during FLIP. Error bars are standard error of the mean (N = 9 for ctrl miRNA; N = 7 for unbleached Golgis and N = 6 for Scyl1 miRNA). No significant difference in the FLIP of Mannosidase II-GFP was observed between ctrl and Scyl1 miRNA expressing cells. For the FRAP experiments (bottom graph) the y-axis = the Golgi fluorescence intensity over the cytosolic fluorescence intensity during FRAP. There was no significance difference in the half maximal recovery time of Mannosidase II in either control of KD conditions. However, p115 KD eliminated Mannosidase II recovery by fragmenting the Golgi. Error bars represent standard error of the mean (ctrl miRNA N = 12; Scyl1 miRNA N = 9; p115 siRNA N = 8).

While our results suggest that the Golgi remains intact following Scyl1 knock down, it is possible that due to limitations in the resolving power of light microscopy this seemingly intact perinuclear Golgi is in fact discontinuous. This was previously shown to be the case for the protein Zeite White 10 (ZW 10), a component of a vesicle-docking complex in the early secretory pathway. Knock down of ZW10 resulted in an expanded Golgi similar in appearance to the one observed in Scyl1 knock down cells, yet fluorescence recovery after photobleaching (FRAP) analysis of a GFP-tagged Golgi resident protein revealed that the Golgi was fragmented [21]. We thus performed FRAP and fluorescence loss in photobleaching (FLIP) analysis on living Hela cells co-transfected with Mannosidase II-GFP (Mann II-GFP) and previously characterized vectors expressing monomeric red fluorescent protein (mRFP) and control or Scyl1-specific inhibitory forms of micro (mi)RNA [16]. Diffusion of MannII-GFP within the Golgi membrane is an established method for assessing the continuity of the Golgi [22]. miRNA expressing cells were identified by mRFP expression, a region of the Golgi was bleached, either repeatedly for FLIP or singly for FRAP, and a sequential time series of images was collected (Figure 3A and Movies S4 and S5). Recovery of MannII-GFP fluorescence in the bleached area during FRAP or depletion of fluorescence outside of the bleached area during FLIP are indicative of diffusion of MannII-GFP throughout the Golgi stack [22]. Numerous cells were analyzed for both protocols revealing that recovery and loss rates were not significantly different between control and knock down conditions, indicating that the Golgi had a similar connectedness between the two groups (Figure 3B). As a control, HeLa cells were transfected with siRNA against p115, which is known to cause Golgi fragmentation. Following p1115 knock down, MannII-GFP did not recover during FRAP analysis (Figure 3, A and B), further validating the results obtained in Scyl1-depleted cells.

**Figure 4:**
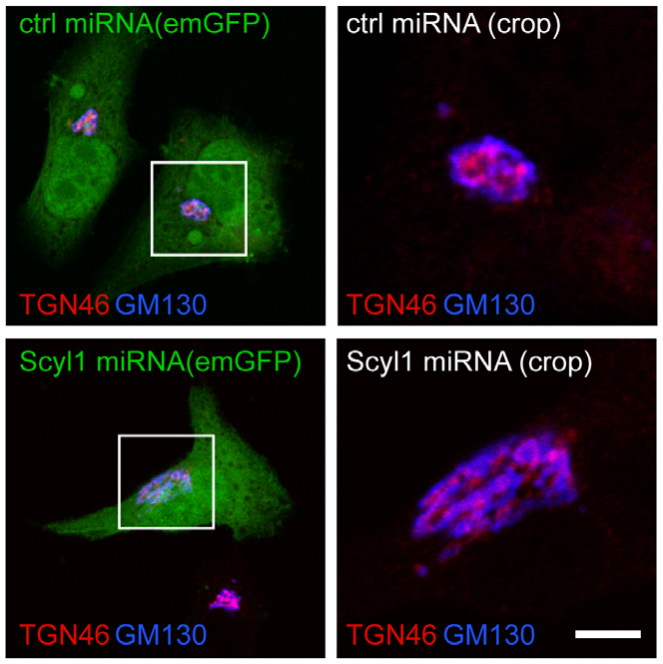
The expanded Golgi remains polarized. HeLa cells were transfected with control (ctrl) or Scyl1 inhibitory miRNAs that were driven from a plasmid that also expresses emGFP. The cells were subsequently fixed and processed for indirect immunofluorescence with antibodies against TGN46 (red) and GM130 (blue). The box in the left panels indicates the expanded area shown in the right panels. The scale bar = 10 µm for the left and 4 µm for the right images, respectively.

To assess if Golgi polarization was intact in Scyl1-depleted cells, vectors expressing emerald GFP (emGFP) and previously characterized control and Scyl1-specific miRNAs [16] were transfected into HeLa cells. Co-staining with TGN46, which is enriched at the TGN at the steady-state and the cis-Golgi localized GM130 revealed that Golgi polarization was intact in both control and Scyl1 knock down conditions, as indicated by the close adjacency but lack of overlap of TGN46 and GM130 (Figure 4).

### EM Analysis of Scyl1-Depleted Cells Reveals Alterations in Golgi Ultrastructure

**Figure 5:**
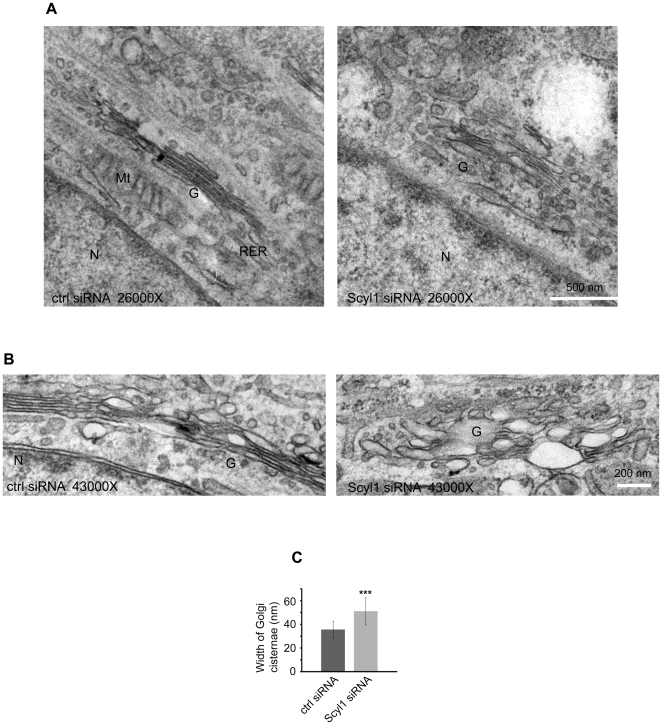
EM analysis of Golgi ultrastructure. **A/B,** HeLa cells were treated with control (ctrl) or Scyl1 siRNA and were then processed for EM analysis. Images show representative Golgi stacks at 26,000X (**A**) and 43,000X (**B**). **C,** The luminal width of the Golgi stacks was quantified.

To examine Golgi ultrastructure following loss of Scyl1, we performed EM analysis on control and Scyl1 knock down HeLa cells. We confirmed efficient knock down and a uniform Golgi expansion phenotype by light level immunofluorescence analysis of parallel cultures prior to the EM analysis. While control cells possessed well-organized perinuclear Golgi stacks, the Golgi apparatus in Scyl1 knock down cells was larger and less organized (Figure 5, A and B). Moreover, the individual stacks were expanded (greater luminal width) (Figure 5, A and B). Quantification of EM images revealed that the increase in luminal width was significant (Figure 5C). Similar changes in luminal width (i.e. swollen cisternae) have been observed following over expression of VSVG protein [3] and knock down of the golgin TMF [23] and may thus result from alterations in membrane flux to and from the Golgi.

**Figure 6:**
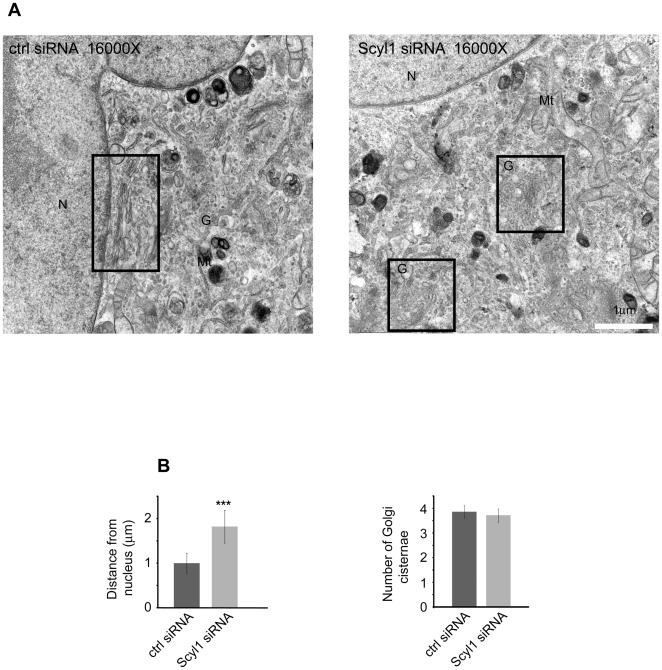
EM analysis of Golgi localization. **A,** HeLa cells were treated with control or Scyl1 siRNA and were then processed for EM analysis. Images show representative cells at 16,000X with the Golgi stacks indicated by black boxes. **B,** The average distance of a Golgi stack from the nucleus and the number of Golgi cisternae were quantified.

In addition, Scyl1 knock down resulted in the Golgi apparatus being on average found further away from the nuclear envelope (Figure 6, A and B). In contrast, the number of Golgi cisternae remained unchanged between control and knock down conditions (Figure 6B). These data are consistent with the phenotype observed at the light level for Scyl1-depleted cells.

### Scyl1 Shares Characteristics of Golgins

**Figure 7:**
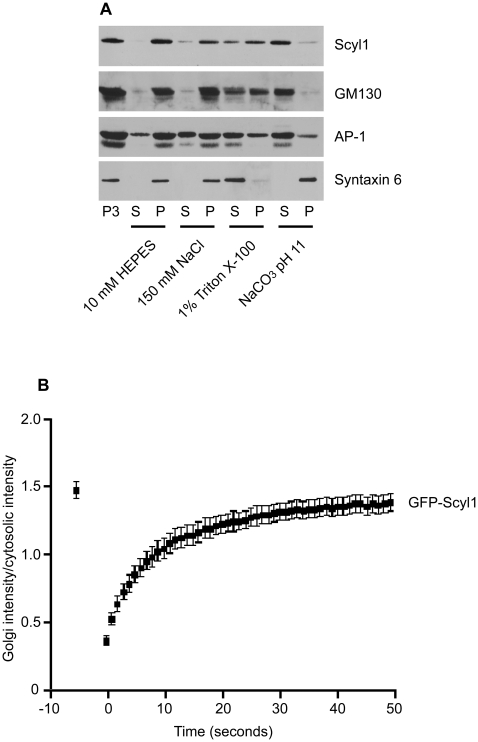
Scyl1 has several properties of golgins. **A,** P3 microsomes, enriched in Golgi membranes, were prepared from brain extracts and were resuspended in 10 mM HEPES, pH 7.4 or the same buffer containing 150 mM NaCl or 1% Triton X-100, or in Na-carbonate buffer at pH 11. The samples were then spun at high g and the supernatant (S) and pellet (P) fractions were analyzed by Western blot with the indicated antibodies. **B,** GFP-Scyl1 was transfected into HeLa cells and the Golgi area was bleached. The recovery of fluorescence on the Golgi was recorded over time.

Proteins are defined as golgins by various cell biological criteria. Scyl1 shares several of these criteria but there are also several important differences. Many golgins are localized to the cis-Golgi and we have previously demonstrated that Scyl1 co-localizes with GM130 and 58K, two cis-Golgi localized proteins [16]. However, Scyl1 is additionally localized to the ERGIC [16]. Furthermore, many golgins are peripheral membrane-associated proteins that possess extensive coiled-coil domains and are resistant to extraction by Triton X-100 [5]. Analysis of Scyl1 primary sequence using the COILS server [24] reveals that the C-terminal region of Scyl1 possesses a coiled-coil domain although it covers three-heptad repeats only (data not shown), unlike most golgins that have coiled-coil regions throughout much of their length. Like golgins, however, Scyl1 is resistant to Triton X-100 extraction from Golgi-enriched membranes, but is extracted by carbonate buffer at pH 11, an identical extraction profile as GM130 (Figure 7A). Comparatively, the transmembrane protein syntaxin 6 is resistant to pH 11 but is extracted by Triton X-100 (Figure 7A).

Golgins are dynamic molecules that cycle on and off the Golgi apparatus, exchanging with their cytosolic pools. To better understand the dynamic nature of Scyl1 we undertook live cell imaging studies of GFP-Scyl1, which was previously shown to have a similar subcellular localization as the endogenous protein [16]. We first calculated the diffusion constant (D) for GFP-Scyl1 using FRAP analysis of the cytosolic pool. We determined a D of 0.37 um^2^/sec (data not shown), which is slower than predicted for a freely diffusing molecule (i.e. ∼15 um^2^/sec). Previous studies looking at the diffusion kinetics of εCOPI-GFP determined a D of ∼0.5 um^2^/sec, similar to our observed value for GFP-Scyl1 [25]. Upon bleaching of the Golgi pool of GFP-Scyl1, we measured a rapid recovery with a t_1/2_ of 5.2 seconds (Figure 7B and Movie S6). Thus, it appears likely that Scyl1 has a dynamic association with the Golgi.

Finally, most golgins have been shown to bind to Rab GTPases and in fact Rab binding has been considered a hallmark feature of golgins [5]. However, we were unable to obtain convincing evidence for Scyl1 binding to either Rab1A in the early secretory pathway or the Golgi-associated Rab6 (data not shown). Binding assays were performed with GST-Rabs under both nucleotide-free and GTP-loaded conditions. We also failed to detect Scyl1 interaction with any of Arf1, 2, 4, or 5, again under GTP-loaded or nucleotide-free conditions (data not shown).

### Scyl1 Interacts with 58K and Requires an Intact Golgin Network for Golgi Localization

**Figure 8:**
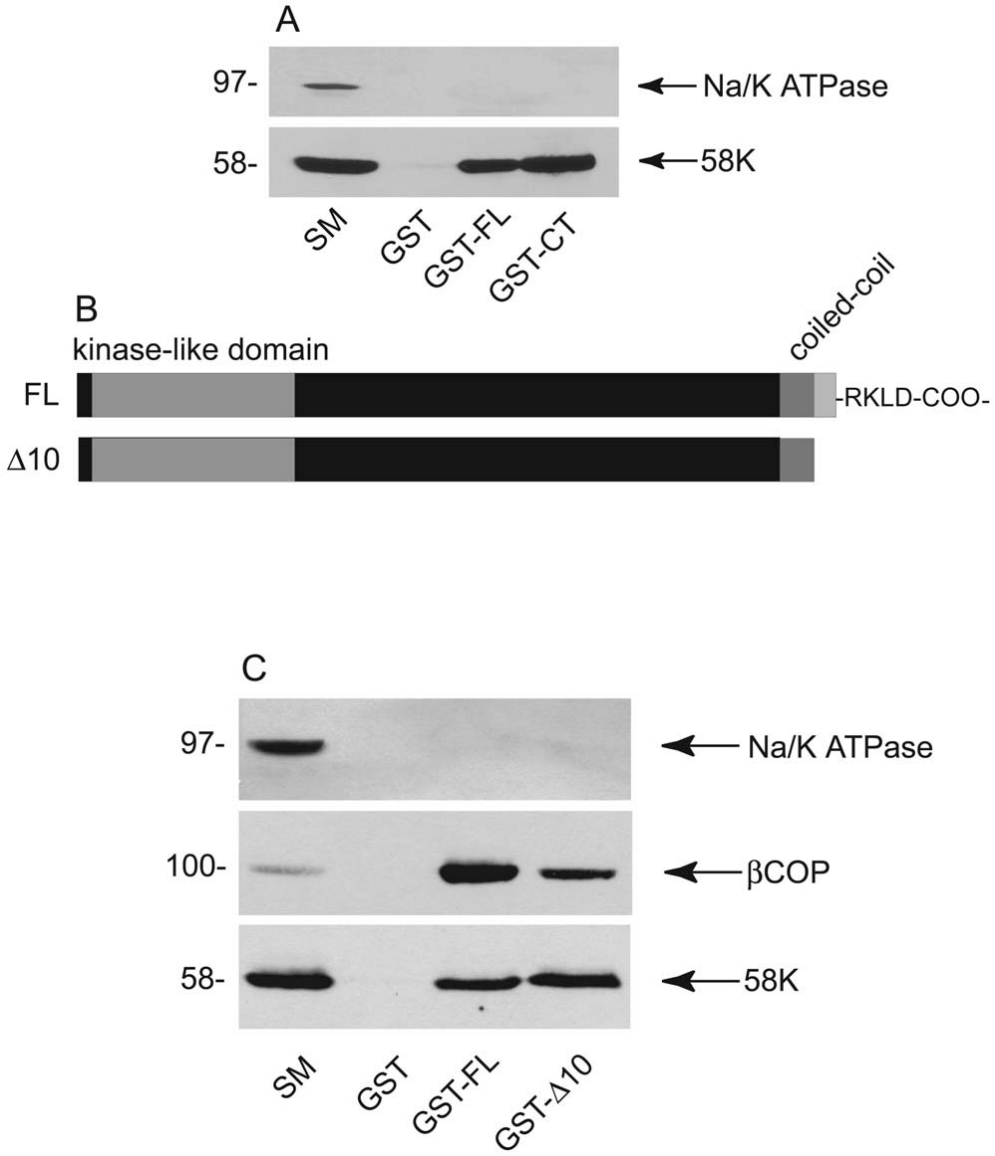
Scyl1 binds 58K. **A,** GST-tagged full-length Scyl1 (GST-FL) or the isolated C-terminal region (GST-CT), along with GST alone were incubated with rat liver extracts and specifically bound proteins were processed for Western blot with antibodies against Na/K ATPase and 58K. **B,** Schematic representation of Scyl1 full-length (FL) and the full-length protein lacking the last 10 amino acids (Δ10). **C,** The constructs described in **B** were used in affinity selection assays followed by Western blot with antibodies for Na/K ATPase, 58K and βCOP. For **A** and **C**, starting material (SM) represents 1/20 of the input to the selection experiment.

Despite the fact that Scyl1 does not appear to be a golgin per se, the fact that it co-localizes with golgins on the cis-Golgi and is resistant to extraction with Triton X-100 suggests that it may interface with the golgin network. Interestingly, while searching for potential Scyl1-binding partners, we identified 58K, which binds to full-length Scyl1 and equally well to the isolated C-terminal region of Scyl1 (Fig. 8A). This is consistent with the close co-localization of endogenous 58K and Scyl1 [16]. In contrast, GM130 did not bind to Scyl1 (data not shown).

Scyl1 ends with the sequence RKLD-COO^−^ (Figure 8B), similar to the COPI-binding dilysine motif, KKXX-COO^−^ and we previously demonstrated that deletion of the last 10 amino acids of Scyl1 (Scyl1Δ10) greatly reduces binding to COPI proteins [16]. Interestingly, we now demonstrate that whereas Scyl1Δ10 (Figure 8B) has strongly reduced binding to βCOP, binding to 58K is unchanged (Figure 8C). Thus, the major sites for βCOP and 58K binding within Scyl1 are non-overlapping, suggesting that Scyl1 could simultaneously interface with both proteins.

**Figure 9:**
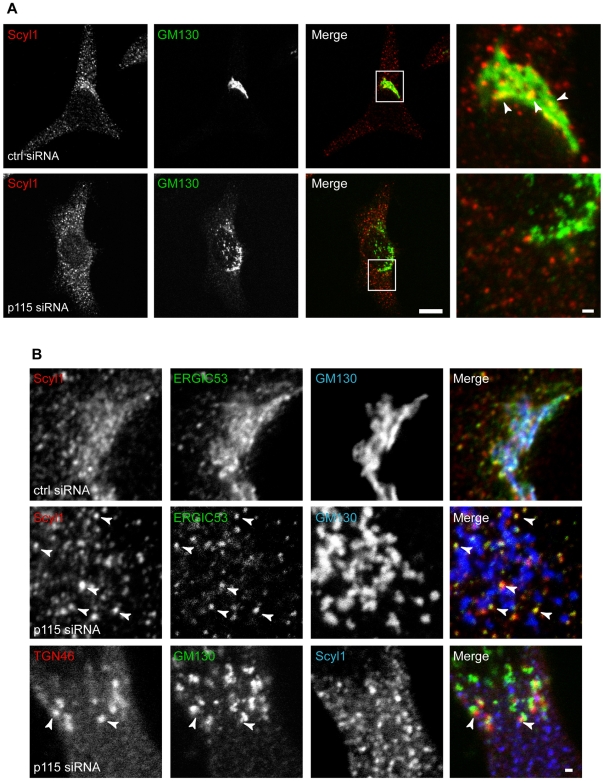
Disruption of the golgin network displaces Scyl1 from the Golgi. **A,** HeLa cells were transfected with control or p115 siRNA and were subsequently fixed and processed for indirect immunofluorescence with antibodies against Scyl1 (red) and GM130 (green). The area indicated by the box in the merged image is shown at higher power on the right. Arrowheads indicate co-localizing structures. The scale bar = 10 µm for the low and 1 µm for high power images, respectively. **B,** HeLa cells were transfected with control or p115 siRNA and with ERGIC53-YFP, and were subsequently fixed and processed for indirect immunofluorescence with antibodies against Scyl1 (red) and GM130 (blue) with ERGIC53 imaged in the green channel; top eight panels or with antibodies against TGN46 (red), GM130 (green) and Scyl1 (blue); bottom 4 panels. For the middle four panels, arrowheads indicate co-localizing structures. For the bottom four panels, arrowheads indicate areas where TGN46 and GM130 are found in close apposition and lack the presence of Scyl1. The scale bar = 1 µm.

To further test the hypothesis that an intact golgin network is required for Scyl1 localization, we sought to destabilize this network in HeLa cells by depleting p115 by RNAi. HeLa cells were transfected with a previously characterized siRNA against p115, which has been shown to result in Golgi fragmentation [20] (Figure 2C). Quantification of p115 siRNA-treated cells revealed that ∼90% of these cells had a fragmented Golgi, as determined by GM130 staining (data not shown). Interestingly, Scyl1 was lost from the GM130-positive Golgi membranes in p115-depleted cells (Figure 9A). In contrast, TGN46 remained in close apposition with GM130-positive Golgi fragments (Figure 9B). The loss of Scyl1 from the Golgi following p115 knock down is in sharp contrast to what we previously observed following other treatments that disrupt the Golgi. Specifically, following treatment of cells with brefeldin A (BFA), which causes the collapse of the Golgi into the ER, and the formation of Golgi remnants, Scyl1 and GM130 remain co-localized on the Golgi remnants [16], [26]. Also, Scyl1 remains co-localized with GM130 and 58K on Golgi ministacks generated by the treatment of cells with the microtubule depolymerizing drug nocodozole [16], [17], [27]. Thus, it appears that an intact golgin network is required for the localization of Scyl1 to the Golgi. Despite the loss of Scyl1 from Golgi membranes following golgin disruption, a pool of Scyl1 remains punctate and in fact continues to co-localize with ERGIC53 on ERGIC membranes (Figure 9B). These data support the hypothesis that Scyl1 is associated with the cis-Golgi via interactions with 58K and the golgin network.

## Discussion

We previously identified Scyl1 as a high affinity-binding partner for COPI coats, and determined a role for Scyl1 as a regulatory component in COPI-mediated retrograde trafficking of KDEL receptor [16]. Here we demonstrate that depletion of Scyl1 results in an expanded but intact Golgi apparatus. A similar Golgi expansion phenotype was seen in NRK cells following knock down of TMF/ARA160, a Rab6-binding protein that localizes to the Golgi [23]. In this case, there was a loss of the tight, juxtanuclear Golgi staining with a modest dispersal of the Golgi, which appeared to remain intact [23]. Combined with our previous work, our current study supports the hypothesis that a decrease in retrograde flux of KDEL receptor-positive COPI vesicles following Scyl1 knock down increases the size of the Golgi, similar to the increase in size observed following an increase in anterograde trafficking flux upon VSVG overexpression [3]. It is known that COPI function is essential in cells, and that lack of εCOP leads to cell death, while knock down of βCOP disrupts Golgi homeostasis [9], [10]. It is less clear how depletion of COPI accessory factors influence cellular function. Loss of function of Scyl1 results in neurodegeneration of motor neurons in mdf mice, resulting in a recessive spinocerebellar ataxic disorder [15], [28]. This suggests that loss of a ubiquitously expressed regulatory component of the COPI pathway does not necessarily result in death, but rather selective degeneration of a subset of vulnerable motor neurons that likely are more dependent on the COPI pathway than other cells, perhaps due to their large size and high degree of polarization [29]. Future studies will examine the role of Scyl1 in neuronal Golgi function.

While Scyl1 displays some biochemical properties of a golgin, important differences exist. One property shared by many golgins is that they interact with Rab proteins. We have thus far been unable to detect a convincing interaction of Scyl1 with Rabs. However, previous studies identified the protein Scyl1 binding partner 1 (Scyl1BP1) based on its binding to Scyl1 [30]. Recent evidence demonstrates that Scyl1BP1 (also known as Golgi-associated Rab-binding protein or GORAB) binds Rab6 in a GTP-dependant manner and localizes to the Golgi, prompting the authors to classify GORAB as a golgin [31]. Interestingly, when mutated, GORAB results in Gerodermia Osteodysplastica (GO) in humans [31], [32]. Thus a Scyl1/GORAB/Rab6 complex may exist. However, whereas Scyl1 is preferentially localized to the cis-Golgi, GORAB and Rab6 are polarized to the TGN [16], [31]. Moreover, GO patients suffer from degeneration of bone and skin but do not appear to display neurological deficits or neurodegeneration [31]. Thus, whether or not Scyl1 has direct or indirect interactions with Rabs is at present unknown.

We did detect an interaction of Scyl1 with 58K and we demonstrate that an intact golgin network is required for Scyl1 association with the Golgi. 58K is a Golgi localized protein that appears to mediate an interface between the Golgi and the vimentin intermediate filament cytoskeleton [33]. As for Scyl1, 58K is tightly associated with Golgi membranes but cycles on and off the Golgi [17]. Moreover, like Scyl1 58K remains associated with Golgi fragments during microtubule disruption and is not released into cytosol during BFA treatment [16], [17]. Thus, it is possible that 58K contributes to Scyl1 recruitment to the Golgi. Since 58K and βCOP bind to predominantly non-overlapping sites on Scyl1, Scyl1 could be at an interface between 58K, the golgin network and COPI coats. Disruption of the golgin network or knock down of Scyl1 would thus disrupt retrograde COPI-mediated trafficking.

Another important property of golgins is that in many cases, for example with GM130 and p115, their knock down results in a fragmented Golgi. In contrast, Scyl1 knock down cells maintain an intact and polarized Golgi as shown from the polarized relationship between GM130 and TGN46, as well as by EM analysis of Scyl1-depleted cells. Thus, unlike GM130, Scyl1 is unlikely to have a role in tethering adjacent Golgi cisternae [34]. It is known that p115, Giantin and GM130 function as tethers to mediate vesicle-docking events at the cis-Golgi. It is hypothesized that only incoming vesicles are affected by these docking events, and that retrograde trafficking should be relatively unperturbed [5]. However, it has been shown that p115-positive COPI vesicles contain KDEL receptor [13], and we have shown that knock down of Scyl1 results in a defect of trafficking of the KDEL receptor from the cis-Golgi to the ER [16]. Moreover, knock down of p115 disrupts the localization of Scyl1 on the Golgi. Therefore, it is possible that Scyl1 uses its 58K binding/golgin network association to position itself to regulate KDEL receptor trafficking events more directly, possibly through the modulation of KDEL receptor phosphorylation, a state that is known to be critical for KDEL receptor inclusion in COPI retrograde directed vesicles [35]. Although the N-terminal kinase-like domain of Scyl1 almost certainly lacks catalytic activity, it could function as a kinase competitor or substrate trap to regulate the phosphorylation status of proteins. In conclusion, our data demonstrate that Scyl1 has a unique position between the golgin network and the COPI machinery and that it regulates Golgi homeostasis, most likely via the modulation of retrograde membrane trafficking events.

## Materials and Methods

### Ethics Statement

The animal experiments in this study were performed under a protocol approved by the Montreal Neurological Institute Animal Care Committee in compliance with guidelines established by the Canadian Council on Animal Care.

### Antibodies and Constructs

A previously characterized and validated affinity-purified rabbit polyclonal antibody against mouse Scyl1 (aa 778-806) was a generous gift from Dr. Gustav Lienhard (Dartmouth Medical School) [16], [36]. Monoclonal antibodies against GM130 and the γ-adaptin subunit of adaptor protein 1 (AP-1) were from BD Transduction Laboratories. Monoclonal antibodies against 58K and the Na^+^/K^+^-ATPase were from Sigma and Upstate Biotechnology, respectively. Monoclonal antibody against βCOP used for immunofluorescence was a gift from Dr. John Bergeron (McGill University) [37] and monoclonal antibody against βCOP used for Western blot was from AbCam. A sheep antibody for TGN46 was from Serotec. An antibody against Syntaxin 6 was a gift from Dr. Richard Schellar (Genentech Inc.). Mannosidase II fused to GFP at its C-terminus (MannII-GFP) was a gift from Dr. John Presley and was previously described [38]. ERGIC53-YFP, GFP-Scyl1, GST-Scyl1 and GST-Scyl1Δ10 constructs were previously described [16].

### Inhibitory RNA-Mediated Knock Down

Knock down of Scyl1 using two 21-nucleotide siRNA sequences was previously described and validated [16]. Knock down of p115 with the siRNA (5′-AAGACCGGCAATTGTAGTACT-3′) was performed as for Scyl1 [16]. The p115 sequence was previously characterized and validated [20]. The production and use of control and Scyl1-specific miRNA sequences in a mRFP vector was previously described [16]. The miRNA expression vector pcDNA6.2/GW-emGFP-miR was from Invitrogen, and previously validated control and Scyl1-specific miRNA sequences were cloned into the vector as described for the mRFP vector [16].

### Electron Microscopy (EM)

HeLa cell monolayers were washed in 0.1 M Na cacodylate buffer (Electron Microscopy Sciences) and fixed in 2.5% glutaraldehyde (Electron Microscopy Sciences) in Na cacodylate buffer overnight at 4°C. The following day the cells were washed in 0.1 M Na cacodylate buffer and incubated in 1% osmium tetroxide (Mecalab) for 1 h at 4°C. The cells were dehydrated in a graded series of ethanol/deionized water solutions from 50%–100%. The cells were then infiltrated with a 1∶1 and 3∶1 Epon 812 (Mecalab) ∶ethanol mixture, each for 30 min, followed by 100% Epon 812 for 1 h, transferred into flat moulds, and polymerized overnight in an oven at 60°C. The polymerized blocks were trimmed and 100 nm ultrathin sections cut with an UltraCut E ultramicrotome (Reichert Jung) and transferred onto 200-mesh Cu grids (Electron Microscopy Sciences) having formvar support film. Sections were post-stained for 8 min with 4% uranyl acetate (Electron Microscopy Sciences) and 5 min with lead citrate (Fisher Scientific). Samples were imaged with a FEI Tecnai 12 transmission electron microscope (FEI Company) operating at an accelerating voltage of 120 kV and equipped with a Gatan 792 Bioscan 1k×1k Wide Angle Multiscan CCD Camera (Gatan, Inc.). Quantification of Golgi morphology was performed on the original *.dm3 files obtained during EM imaging using the line measure tool in Image J software 1.4.2c (National Institutes of Health, Bethesda, MD) (NCBI) [39].

### Immunofluoresence

Cells were fixed in cold 3% paraformaldehyde at 4°C for 20 min, and washed in cold PBS. Cells were then permiabilized in 0.2% Triton X-100 (in PBS) for 4 min, washed with PBS, and then incubated in 1% bovine serum albumin (BSA) in PBS for 30 min, and primary antibodies in 1% BSA were added and incubated on the cells either overnight at 4°C or 2 h at room temperature. Cells were then washed with 1% BSA and secondary antibodies were added for 1 h at room temperature. The cells were then washed in 1% BSA, with a final wash in PBS, before mounting on coverslips with mounting media (Dako Inc.). Cells were imaged on a Zeiss 510 confocal microscope using an oil immersion 63X objective with a NA of 1.4.

### Quantification of Golgi Surface Area

To calculate the two-dimensional surface area of the Golgi, images were analyzed and quantified in Image J 1.42c (National Institutes of Health, Bethesda, MD) [39]. LSM files were opened and the area of the Golgi apparatus outlined manually, and the two-dimensional surface area calculated with the measure tool in Image J. For GM130 analysis of surface area; control siRNA (N = 3; n = 263), for Scyl1 siRNA #1 (N = 3; n = 325), for Scyl1 siRNA #2 (N = 3; n = 300), for 58K analysis of surface area; control siRNA (N = 3, n = 108), for Scyl1 siRNA #1 (N = 3; n = 200) were analyzed. For quantification of (Golgi surface area)/(total cell surface area) 98 cells were measured for control conditions and 88 for knock down conditions.

### Three-Dimensional Rendering and Quantification of Golgi Stacks Using Imaris (Bitplane Inc.) Software

All HeLa cell Z-series reconstructions were imaged with a pinhole size of 1 Airy Unit with a Z-step interval of 0.2 µm at 4x zoom. For analysis of control and Scyl1-specific siRNA treated cells the same confocal settings were maintained between coverslips from the same experiment. HeLa cells transfected with control (N = 2, n = 27) or Scyl1 #1 (N = 3, n = 27) siRNA, were stained with GM130 and imaged. Students t-test revealed a significant difference between the volume of the Golgi for control and knock down conditions (p<0.001). For these, and identical experiments in which HeLa cells were stained for βCOP (control N = 3, n = 22; Scyl1 #1: N = 3, n = 13; p<0.001), LSM Z-series files were opened in Imaris 5.7.2 as volumes and then isosurfaced using a 0.2 µm Gaussian Filter. Each Golgi isosurfacing was visually inspected to ensure that it followed closely to the underlying original three-dimensional image and did not incorporate voxels outside of the observable Golgi region. Each isosurfaced Golgi was selected and the volume statistic recorded.

### Live Cell Imaging

Fluorescence recovery after photobleaching (FRAP) and fluorescence loss in photobleaching (FLIP) experiments were performed at 37°C on a Zeiss LSM 510 inverted microscope with a 63X/1.4 NA oil immersion objective. On the day of imaging, media was replaced with HBSS supplemented with 25 mM HEPES and 4 g/L D-glucose and live imaging experiments were conducted in this media. HeLa cells plated on 35 mm live imaging glass bottom petri dishes (Mattek Corp.) were co-transfected with the Golgi resident transmembrane glycoslytransferase MannII-GFP and either control or Scyl1-specific miRNA vectors that co-express mRFP. After 4 days of expression, to allow for effective knock down of Scyl1, the steady-state distribution of MannII-GFP at the Golgi was confirmed. For other experiments, cells were transfected with a previously verified GFP-Scyl1 construct [16]. For all live cell experiments, cells were imaged at low resolution (8 bit mode; 512×512 pixels; 1x averaging (4x averaging was used for MannII-GFP kinetic analysis); 1 second/frame, 2x zoom). Laser settings and general procedures were based on Cole et al. [22]. Briefly, 100% laser intensity was used for bleaching; 5-10% laser intensity for acquisition; 50-60 iterations/bleach, and a pinhole setting of 2.89 Airy units corresponding to a 2 µm optical slice. LSM time series files were analyzed with Image J 1.42c using the time series analyzer plug-in (National Institutes of Health, Bethesda, MD and Dr. Balaji (UCLA)).

For quantification of GFP-Scyl1 recovery following bleaching of the Golgi, the ratio of (background subtracted Golgi intensity)/(background subtracted cytosolic intensity) was plotted through time. Half maximal recovery intensity (t_1/2_) was calculated as the ((average intensity of the plateau) + (postbleach value))/2, and the time corresponding to this intensity value determined from manual inspection of the raw data. N = 3, n = 13 cells were analyzed. To calculate the Scyl1 diffusion coefficient in the cytosol, the circle ROI tool on the Zeiss 510 was used to denote areas in the cytosol for photobleaching, and recovery image series over time were collected at 1 frame/second (N = 2, n = 15). The equation D = 0.88*(w^2^/4t_1/2_) was used to calculate the diffusion coefficient for cytosolic Scyl1, where w = the radius of the circle ROI used during photobleaching, and t1/2 was the time of half maximal fluorescence recovery of GFP-Scyl1.

For FRAP analysis of MannII-GFP, mean intensity values of fluorescence recovery were background subtracted and normalized to the pre-bleach intensity value of the Golgi and plotted as a percent of this value over time. When non-bleached Golgis were present in the same field they were also measured and used as an internal control for focal plane drift and light scattering. To control for contributions from the ER of ManII-GFP we conducted control experiments in which the entire Golgi apparatus was bleached, and recovery measured over time. No detectable fluorescence recovery was detected in Golgis that were completely bleached over the time scales used in our experiments (data not shown). Finally, to control for light scatter and non-specific bleaching outside of the target area fixed specimens were photobleached using similar conditions as those described above, and bleaching was not observed outside the target area (data not shown). For Scyl1 miRNA (N = 3, n = 9) and control miRNA (N = 3, n = 12) cells were measured. For FRAP analysis of MannII-GFP in p115 siRNA transfected cells, methods were followed as described above.

### Membrane Extraction Profile of Scyl1

Four rat brains were homogenized in 10 mM HEPES buffer, pH 7.4 with protease inhibitors (0.83 mM benzamidine, 0.23 mM phenylmethylsulfonyl fluoride, 0.5 µg/ml aprotinin, and 0.5 µg/ml leupeptin) (Buffer A) +0.3 M sucrose with 9 strokes of a glass-Teflon homogenizer. The homogenate was diluted to a total volume of 40 ml and spun at 750×g for 5 min. The supernatant from this spin was spun again at 12 000×g for 15 min. The supernatant was taken again and spun at 180 000×g for 30 min and the pellet from this spin (P3) was resuspended in ice cold Buffer A. The resuspended pellet was diluted 1∶10 with Buffer A to a final concentration of 2 µg/µl. 800 µl samples were aliquoted and spun at 400 000×g for 15 min. The resulting pellets were resuspened either in Buffer A, Buffer A +150 mM NaCl, Buffer A +1% Triton X-100 or Buffer A +50 mM NaCO_3_ pH 11.0. The samples were then incubated on ice for 15 min, and spun again at 400 000×g for 15 min. Aliquots of 100 µl from both the supernatant and resulting pellets were resuspended in their respective buffers and loaded for analysis by SDS-PAGE.

### Affinity Selection Assays

Rat livers were homogenized in buffer A, spun at 800×g for 5 min, the supernatant collected and Triton X-100 was added to 1% final. Following 15 min at 4°C, the extracts were spun at 116 000×g for 30 min and the supernatant was adjusted to 1 mg/ml and NaCl was added to 50 mM final. Aliquots of 1 ml were incubated for 3 h at 4°C with GST or GST-Scyl1 fusion proteins pre-coupled to glutathione-Sepharose beads. Beads were subsequently washed 3 times with ice cold buffer A with 1% Triton X-100 and 50 mM NaCl, eluted in SDS-PAGE sample buffer and processed for Western blot.

## Supporting Information

Movie S1 Expansion of the Golgi following Scyl1 knock down. HeLa cells treated with ctrl siRNA or Scyl1 #1 siRNA were subsequently mixed and co-stained for Scyl1 (red) and 58K (green). A projection of the cells from a confocal stack generated from a Z-series is displayed. A single image from the same stack is presented in Fig. 3C. The two cells in the middle lack Scyl1 and have an expanded Golgi.
(0.49 MB AVI)

Movie S2 Three-dimensional projection of a Golgi from a control siRNA-treated cell. HeLa cells transfected with control siRNA were stained for GM130. A Z-series of a complete Golgi stack was imaged by confocal microscopy and volume surface rendered using Imaris software. A single image from this projection is displayed in Fig. 2A.
(0.95 MB AVI)

Movie S3 Three-dimensional projection of a Golgi from a Scyl1 siRNA-treated cell. HeLa cells transfected with Scyl1 siRNA were stained for GM130. A Z-series of a complete Golgi stack was imaged by confocal microscopy and volume surface rendered using Imaris software. A single image from this projection is displayed in Fig. 2A.
(1.73 MB AVI)

Movie S4 FRAP analysis of a Golgi from a Scyl1 miRNA-treated cell. HeLa cells transfected with Scyl1 miRNA and MannII-GFP were subjected to FRAP analysis as described in the Materials and Methods. The image reveals the MannII-GFP fluorescence. The Golgi on the right was photobleached on the left side and fluorescence recovery was monitored over time. Selected images from this movie are displayed in Fig. 3A.
(0.13 MB AVI)

Movie S5 FLIP analysis of a Golgi from a Scyl1 miRNA-treated cell. HeLa cells transfected with Scyl1 miRNA and MannII-GFP were subjected to FLIP analysis as described in the Materials and Methods. The image reveals the MannII-GFP fluorescence. The Golgi was photobleached in the middle continuously through the time of the movie. The scale bar = 10 µm.
(0.19 MB AVI)

Movie S6 FRAP analysis of GFP-Scyl1. GFP-Scyl1 was transfected into HeLa cells and the Golgi area was bleached. The movie records the recovery of fluorescence on the Golgi over time.
(1.10 MB AVI)
